# Nurses' Experience of Using an Application to Support New Parents after Early Discharge: An Intervention Study

**DOI:** 10.1155/2015/851803

**Published:** 2015-01-28

**Authors:** Dorthe Boe Danbjørg, Lis Wagner, Bjarne Rønde Kristensen, Jane Clemensen

**Affiliations:** ^1^Research Unit of Nursing, Institute of Clinical Research, University of Southern Denmark, Campusvej 55, 5230 Odense M, Denmark; ^2^Odense University Hospital, Department of Gynaecology and Obstetrics, Søndre Boulevard 29, 5000 Odense C, Denmark; ^3^Odense University Hospital, CIMT, University of Southern Denmark, Søndre Boulevard 29, 5000 Odense C, Denmark

## Abstract

*Background.* A development towards earlier postnatal discharge presents a challenge to find new ways to provide information and support to families. A possibility is the use of telemedicine.* Objective.* To explore how using an app in nursing practice affects the nurses' ability to offer support and information to postnatal mothers who are discharged early and their families.* Design.* Participatory design. An app with a chat, a knowledgebase, and automated messages was tried out between hospital and parents at home.* Settings. *The intervention took place on a postnatal ward with approximately 1,000 births a year.* Participants.* At the onset of the intervention, 17 nurses, all women, were working on the ward. At the end of the intervention, 16 nurses were employed, all women.* Methods.* Participant observation and two focus group interviews. The data analysis was inspired by systematic text condensation.* Results.* The nurses on the postnatal ward consider that the use of the app gives families easier access to timely information and support.* Conclusions.* The app gives the nurses the possibility to offer support and information to the parents being early discharged. The app is experienced as a lifeline that connects the homes of the new parents with the hospital.

## 1. Background

Since the 1990s, the average length of postnatal hospital stay has declined, both in Denmark and internationally. The most prominent reasons are a renewed focus on the fact that giving birth is not a disease and the general need for cost savings in the healthcare system [1–4]. In Denmark, the average length of postnatal hospitalization has decreased from 92 hours in 2007 to 77 hours in 2012 [3].

A Danish questionnaire study (*N* = 1, 507 women) identified that 44.3% of the women who were discharged early (within 24 hours) from postnatal care experienced a lack of follow-up support; that is, they felt that they did not receive the support needed to care for the newborn; 37.5% did not receive support for postnatal self-care, and 46.1% did not receive adequate support around breastfeeding [5]. These findings concur with results in international research [2, 6, 7].

Studies show that new parents experience concerns, uncertainty, doubts, and feelings of insecurity during the postnatal period and are in need of follow-up support after early discharge [2, 6, 8–10]. Support is important when becoming a parent—Barclay et al. underline that one of the mediating factors in becoming a mother is “the nature of social support available,” which includes partner, family, friends, and health professionals [11].

A sense of security is a central element to support as it might influence a parent's journey towards becoming a successful parent. Persson et al. have developed the concept “parents' postnatal sense of security.” They identified the following dimensions as important for both parents' postnatal sense of security: empowerment from staff, affinity within the family, and the health and wellbeing of the family. An empowering organisation was fundamental for strengthening this [9, 12–15].

If the parents feel insecure it can have a negative effect on parental self-efficacy (PSE). The definition of PSE is as follows: “beliefs or judgments a parent holds of their capabilities to organize and execute a set of tasks related to parenting a child” [16]. For parents to employ parenting behavior positively, they must have confidence in performing the specific behavior. Parents with high self-efficacy are likely to make a greater effort than parents with low self-efficacy. Bandura has clarified what it is that enables an individual to build self-efficacy beliefs. Important aspects are mastery learning, where you can gain positive experiences, when you are doing things yourselves, vicarious experiences, that is, seeing others perform, and verbal persuasion, where others assure you that you hold the ability to perform a certain task [17, 18].

The new trend towards shorter hospital stays has affected healthcare professionals' practice. They experience that they have too little time to support new parents and to give individualised and timely information [6, 19].

In 2011, The Region of Southern Denmark issued a new policy regarding the postnatal period, in which early postnatal discharge (i.e., from four to six hours; max. 24 hours) was to become general practice following uncomplicated delivery for first-time and multiparous mothers. This shift in the postnatal care presents a challenge in terms of finding new ways to provide the sufficient support that meet the needs of the new parents with a postnatal follow-up that can enhance PSE and a sense of postnatal security.

One possibility is the use of telemedicine, which can provide an innovative solution [20–22].

Telemedicine has also been developed within obstetrics practice [23–27]. It seems that telemedicine has the potential to provide appropriate support to early discharged mothers and their families, because it offers the possibility for new parents to be guided by healthcare professionals in their transition into parenthood. Findings by Lindberg show that both parents and healthcare professionals find that telemedicine has the potential to provide appropriate support because it presents new ways to communicate that can substitute for face-to-face contact and it can be a valuable and functional complement to usual practice [25, 26].

We wanted to explore this potential and therefore designed and developed a software application (app), which was tested in a pilot study prior to the intervention [28].

### 1.1. Aim

The aim is to explore how nurses experience using an app in nursing practice and how it impacts their ability to offer support and information to postnatal mothers who are discharged early and their families, in a way that will enhance the families' sense of security and self-efficacy.

## 2. Methods, Participants, and Data Collection

### 2.1. Design

This study applied a participatory design (PD). It combines the use of qualitative methods and intervention, based on collaboration with users. The PD approach involves defining problems and indicating solutions in designing sustainable IT solutions for practice together with the users. An essential aspect of designing and developing a new technology is the intervention phase, where the actual technology is tried out in practice and concrete experiences with the use of the new technology are gained. Participatory design can be viewed as hermeneutics, where new understanding is developed through a circular collaboration between the researcher's understanding and an attempt to interpret a certain phenomenon in collaboration with the participants [29].

PD has its origins in action research [29–32]. Action research spans a wide landscape of differentiated, but primarily qualitative, research strategies for bringing about change through action, developing and improving practice [33].

### 2.2. Intervention

This study was an intervention study where an app was tested between hospital staff and new parents at home following early postnatal discharge. The content, format, and style of the app were designed on the basis of the parents' identified needs, in close cooperation with the nurses on the postnatal ward, and with the assistance of a team of computer programmers. The identified needs have previously been reported in depth [6].

In brief, new families requested an individualised postnatal follow-up, timely information and guidance, and accessibility to, and new ways to communicate with, healthcare professionals. This reflected the professional concern that the nurses had as to how they can ensure a postnatal care, which will ensure a sense of security, wellbeing, and parental self-efficacy, when the new parents are being early discharged. The app was designed with the following functionalities that should accommodate the needs of the early discharged parents.

(1) Asynchronous communication, online chat, where the families could send text messages to the healthcare professionals as well as photos and videos and receive an answer within four hours. This method of communication may diminish the barrier in accessing healthcare professionals after hospital discharge.
(2) A knowledgebase consisting of information material with a search function for easier access to information. The information material was evidence-based and written and compiled by the nurses on the ward. The information material consisted of written material about the postnatal period, for instance, information about breastfeeding, skin-to-skin contact, the mother's restitution after giving birth, and practical advice about baby care. The knowledgebase also contained instructions videos with guidance about breastfeeding, skin-to-skin contact, the wellbeing of the baby, baby clues, and how to bathe the baby.
(3) Messages sent out automatically every 12 hours from the time of birth. The messages relate to the age of the baby and should be relevant to the new parents providing them with information about breastfeeding, the baby's first bowel movement, and so on. The nurses had written down what they would normally inform and instruct the new parents about in the first postnatal days. It was rewritten into short messages that the new families would receive every 12th hour for the first 4 days after their baby was born. In the messages there are relevant links to the knowledgebase with more thorough information. The following is an excerpt of a message.

**Figure 1:**
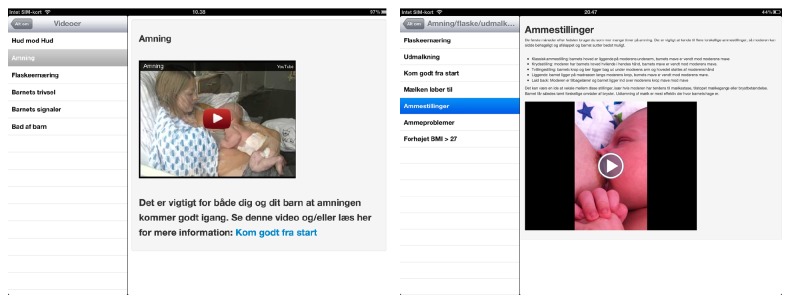


**Figure 2:**
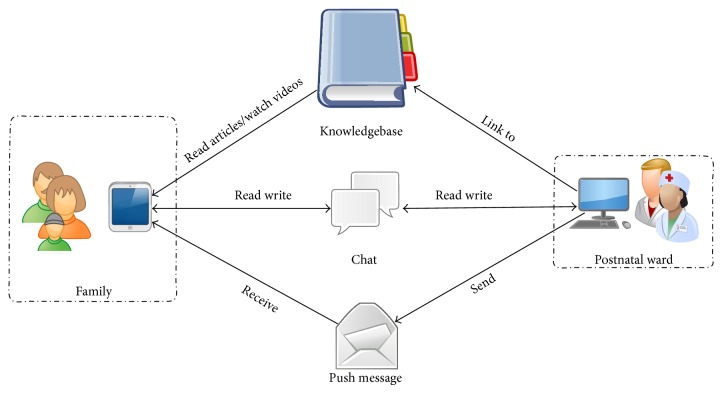


 
*24 hours after giving birth. Your boy has to suck efficiently at least 6–8 times a day. Your baby will often wake up and show signs of hunger, if not you [sic] have to wake him up, read more about that here: “Get a good beginning” and “Breastfeeding.”* (Figures 1 and 2).

The parents were given an iPad to take home on loan on which the app was installed. They had access to the app for seven days. They were to return the iPad to the hospital after seven days in a prestamped package.

Prior to the intervention, we tested the app in a pilot study [28], where the nurses were instructed in the use of the app and the accompanying website. The nurses registered the new parents on the website and used it to check for messages. The nurses were responsible for the online chat, which in practice meant that they had to check it every four hours and send replies to the families. Two of the nurses were responsible for updating the knowledgebase. These responsibilities were additional to the nurses' assigned duties involving caring for the patients admitted to the postnatal ward. No extra time was allocated in their shift for the additional work involved in answering messages.

### 2.3. Sample and Context

The study took place on a postnatal ward that handles approximately 1,000 births a year and included nurses employed on the ward. The management at the ward had initiated the project after the implementation of the new postnatal policy in The Region of Southern Denmark. The nurses at the ward were all involved in the project and willing to participate in the intervention.

During the course of the study, four nurses moved job and three were employed. The newly employed nurses were introduced to the intervention. At the onset of the intervention, 17 nurses, all women, were working on the ward. Their professional postnatal experience varied from less than one year to 30 years, with a mean of 10.2 years. At the end of the intervention, there were 16 nurses employed, all women. Their professional postnatal experience varied from under one year to 30 years, with a mean of 7.1 years.

### 2.4. Data Collection

Participant observation was carried out on the postnatal ward from March to August 2013, on average one day a week, in all 20 days. The data were primarily collected during day shifts, though five times were also during evening shifts. The nurses were not followed through an entire shift, because the focus was how they experienced using the app in nursing practice and how it affected their ability to offer support and information to postnatal mothers who are discharged early.

The data from the participant observation are based on informal conversations with the nurses. The informal conversations took place during the nurses' coffee or lunch breaks or in the nurses' office. Sometimes they spontaneously started talking about the app, and other times we would ask a question to initiate a talk. Occasionally we were also assisting them with practical advice or help concerning the iPads or the webpage, which automatically led to conversations about the app and how they experienced using it.

Field notes were taken concurrently with a focus on place, participants, and activity. The following served as a guideline for the observations: what happens at the time of observation and what intentions and feelings occur in the situation [34].

We also conducted two focus group interviews [35, 36]. All the nurses on the ward who had taken part in the study were invited to a focus group interview. Nine out of a possible 13 nurses attended. The other nurses could not attend on the given dates, due to either work or personal matters. The number of participants who could attend on the chosen dates determined the size of each group, which ended up being four and five. The focus group interviews were held in the employee staff-room on the postnatal ward.

Before each focus group interview commenced, the moderator (the first author) introduced the purpose of the interview and clarified the guidelines and the focus: experiences using the app in nursing practice and how it affects their ability to offer support and information to postnatal mothers who are discharged early.

An interview guide was compiled. The overall theme focused on the nurses' experiences, which formed the basis of the discussion [37, 38]. Some additional questions were asked during the discussion. The development nurse on the ward participated as a comoderator, made notes during the interviews, and evaluated the atmosphere and interaction. The focus group interviews lasted 44 and 55 minutes, respectively, and were audio-recorded and transcribed verbatim.

## 3. Ethical Considerations

The participants received oral and written information about the study and were included after providing their informed consent, in compliance with the Helsinki Declaration [39]. The first author asked the nurses if they would like to participate in a focus group interview, and they were given time to think it over. They were told that participation was voluntary and that the focus group interviews would be held during working hours.

The study was submitted to the Scientific Ethics Committee. The committee decided that approval from an ethics committee was unnecessary according to the national legislation in Denmark (S-20110171). The Danish Data Protection Agency registered and approved the study (2008-58-0035).

## 4. Data Analysis

The data analysis was inspired by Malterud's systematic text condensation (STC) [40] and organised according to the steps taken in the analysis, as shown in Table 1. STC is a descriptive and explorative method used in the analysis of qualitative data, such as interview studies, observational studies, and in the analysis of written texts [41]. Giorgi's psychological phenomenological analysis was the starting point for STC. He developed the descriptive phenomenological method in psychology [38, 41, 42]. STC is a development of Giorgi's principles, including four comparable steps of analysis. It is pragmatic in the sense that it is easy to both follow and share due to the elaborated steps of the analysis.

Firstly, we captured an overall impression of the data and extracted a preliminary set of main themes.

Secondly, the data was divided into meaningful topics, which were relevant to the study question. Next, the meaningful topics were condensed and coded. Finally, the findings were synthesized, involving a shift from condensation to descriptions and categories. The codes were developed based on the preliminary themes identified in the first step and the theoretical framework.

In order to optimise validation, three researchers from the research team were involved in the analysis process. Our findings were subsequently discussed in relation to relevant literature and theory.

## 5. Results

The categories that emerged from the data analysis were as follows:

1. an app as a means of providing support,
2. an app as a means of conveying timely and accessible information.

The categories are presented below and are illustrated by quotations from the two focus group interviews (FGI) and from conversations that took place during the participant observation (PO).

### 5.1. An App as a Means of Providing Support

#### 5.1.1. Adjustment to New Ways of Communicating

The nurses were hesitant at first when they had to chat online with the families, that is, using written instead of verbal communication. The following example occurred during a lunch break on the ward at the very beginning of the intervention.

 
*One of the nurses (nurse T) related that she had answered a message: “Well, I think it was very time consuming. It was all new to me and normally I would just talk on the phone, but I really had to think twice before sending the message”. One of the other nurses (nurse K) supplemented this with: “Yes, it does take quite some time and the mother who wrote, well, how would I put it, the message wasn't well articulated”. Nurse T continued: “It wasn't that it was difficult, but it just felt so different to write to a family instead of just talking”. Nurse K then said: “It is probably also a matter of time—we have to get used to it.”* (Field note, March 2013, PO)

Another concern was that when communicating in writing, one uses fewer of the senses.

 
*I answer their questions (…) I look at the photo of the umbilicus, for instance, or whatever it is. But I do not have the mother's tears. It creates a distance.* (Nurse I, FGI)

Though, after a period of time using the app, the nurses no longer felt that it was such a big challenge or that it involved changes to their work.

 
*Maybe you have to have some ping-pong, to ask the right questions, like you would have asked, if you were in the room [i.e. face to face]. But it hasn't been difficult.* (Nurse D, FGI)

However, they did state that a lot depended on the type of questions that they had to answer on the online chat. Messages that were accompanied by, for example, a photo of an umbilicus were considered “easy” to answer, whereas questions about breastfeeding were more difficult, since more information and dialogue were required in order to make a judgment and give the appropriate support.

 
*But, that's also the point. Well, they can get answers to something very specific, but it is also the intention that where it is very complicated, and there are a lot of problems, we need to see them.* (Nurse A, FGI)

The nurses stressed that the written communication cannot “stand alone,” but they emphasized that there was always the option to invite the parents to come to the ward for more guidance face-to-face and that this occurred on occasion.

The nurses had to check the chat for messages every 4 hours, which showed to be a constant challenge. Explanations given for forgetting to check the online chat were that the nurses were too busy and there were challenges to adjust to the new procedures; the nurses had to go to the office to check the chat, and they usually spend most of their time in the patients' rooms or the nursery room.

 
*We do delegate who is responsible for the chat during the shift, but then oh no we have forgotten it. I have responded to one that was 14 hours old.* (Nurse A, FGI)

#### 5.1.2. Connecting Hospital and Home

The app gave the parents the option to stay at home, while, for instance, having the baby's umbilicus assessed, because they could send a photo. The nurses found that the possibility to send photos was an advantage instead of the parents having to explain how the umbilicus looked like, over the phone. It provided the nurses with a more accurate impression of the umbilicus, and they experienced that it increased their possibility to provide the appropriate advice and support.

The following example shows the differences between the distinctive forms of contact the nurses used. It took place during a coffee break on the ward.

 
*One of the nurses had assessed an umbilicus based on a photo sent using the online chat. She could see that the baby was red in the groin, so she also wrote a note on that to the family. One of the nurses said: “well, that would not be possible over the phone”. To which another replied: “but, if they had been here [on the ward], you could have seen the whole baby, not just the groin, and then you could also check the armpits, for instance.”* (Field note, March 2013, PO)

The nurses agreed that families often found it difficult to contact healthcare professionals, because they did not want to disturb, which they ascribed to cultural factors or general expectations in society.

 
*I also think it is just a cultural thing. Nowadays, people with kids—they want to take care of themselves.* (Nurse D, FGI)

The nurses also discussed that the new parents were reluctant to call the ward for help, even though the nurses told them that they should always call, if they had any doubt when they had been discharged. They thought that it was because the parents had experienced that the nurses were busy, and then they did not want to disturb.

 
*They find it difficult to take contact. They feel it is inconvenient, because they have experienced that we were busy.* (Nurse V, FGI)

The nurses experienced that the app gave the families an opportunity to make contact with them after discharge, where they did not feel that they were intruding.

 
*And I think that's a help. They feel that it is ok that they make contact.* (Nurse V, FGI)

### 5.2. An App as a Means of Conveying Timely and Accessible Information

#### 5.2.1. Accessible Information

The nurses emphasized that one of the advantages of the app was that the information material for the parents was in digital instead of paper form.

 
*Paper, it is all over, a mess, whereas the iPad—they know where that is. It suits them. Paper doesn't.* (Nurse I, FGI)

The nurses expressed that there was a lot of information material handed out at the hospital, and they questioned how much of it the families actually read. They considered it an advantage that it was now in digital form, as it seemed to appeal more to the families, because they could easily access it on the iPad and they could also search within the material in the same way as using “Google” or other search engines.

Another possibility was watching the instruction videos. The nurses experienced that this was a suitable way for the new parents to be guided. For instance, the nurses at the ward showed the admitted parents how to bathe the baby, but this was at a fixed time during the day, and if the parents watched the video, they could watch it whenever they wanted.

 
*When they are admitted for such a short time, it becomes very hectic to tell and show them everything. This way they can do it, when they want to and also when they are at home.* (Nurse K, FGI)

They also found that it was easy for them to refer to a video or a written instruction.

 
*Well she wrote me a question, and I answer her back, but I also wrote that I thought she should read the information, it was easy to do, because I knew that she could find it easily on the app.* (Nurse S, June 2013, PO)

The nurses told that the parents reported that they felt secure with the app. They knew where to look for the information, and at the same time they knew that they could easily get in contact with the nurses at the ward.

 
*And then she [a mother] told me that she was so secure, because it was just like having a nurse standing outside the door.* (Nurse B, August 2013, PO)

#### 5.2.2. Timely Information

The nurses had to adjust to the new policy with the early discharge. It stressed them because they had shorter time with the individual family.

 
*Well they come from the delivery ward, and then they are here for such a short period of time. And they sometimes just fall asleep, when I talk to them. They need something differently.* (Nurse V, FGI)

The nurses expressed that it was reassuring to know that when the families were discharged with the app they were drip-fed information in the form of automated messages. It relieved some the pressure they might feel when discharging mothers early, in terms of the duty to “have informed thoroughly enough.”

 
*I think that there is so much information that they need in such a short time. Then you are just talking and talking, while you think, how much do they remember, when they come home.* (Nurse A, FGI)

The nurses often had a feeling that the families could not retain all the general information. The nurses considered that the automated messages seemed to meet this challenge by providing families with timely information.

 
*Knowing that, if there is something that I have forgotten, they get the pop-up messages, which means they get the information one more time, that's great.* (Nurse A, FGI)

The nurses regarded the automated messages that the families received as a tool to stimulate the families' curiosity and also their capacity to take control of their situation. The nurses believed that because of the interactive links in the automated messages, when the parents read the messages, they could easily read additional information material in the knowledgebase or they could address a question to the nurses on the postnatal ward. The nurses experienced that the parents took control of their situation and the messages made the parents feel well prepared for the postnatal period. The messages served either to reassure them or to allow them to react, if they required more information or support.

 
*It is like a pat on the shoulder. Everything is ok.* (Nurse D, FGI)

## 6. Discussion

In this study, we found that the nurses consider that the app gives them the possibility to offer support to the families discharged early, as it provided easier access to timely information and support, and it enhanced opportunities for families to initiate contact after discharge. They nurses find that the app connects the homes and the hospital.

The nurses state that the written asynchronous environment offers an easy way to offer families support. They feel that it connects the hospital setting with the home and goes some way towards reducing the gap, which families can experience as a barrier, in the fact that they are reluctant to contact the hospital staff for support after discharge [2, 6, 43]. Other studies have also found that when new families are discharged, it is essential that they are able to get professional support whenever they need it [25, 44, 45]. Persson et al. have identified that accessibility to support from healthcare professionals is an essential part of experiencing a postnatal sense of security [9, 14, 15, 46] (Table 2).

The nurses regard the app as a lifeline for families because it increases access to professional support. The app constitutes a new way of making support available. This is in line with the conclusions from a study by Bjoernes et al. in 2012 that explored the possibilities involved in online contact between nurses and men with prostate cancer (*n* = 34). The patients experienced a feeling of partnership in dialogue (via e-mail) that supported their ability to be active and it gave them a feeling of freedom and security. They saw the written asynchronous contact as providing a flexible and calm communication environment and as a way to substitute for the reduction in face-to-face contact at the hospital [47].

Yet an important aspect is that the new parents are depending on the fact that the nurses do check the chat every 4 hours in order to have access to support, and the study showed that it was a constant challenge. Even though the nurses thought they just had to get used to the new routine, we discussed new ways of remembering the chat, because it was critical for the parents' sense of security that they could rely on it.

The nurses in our study also found that when the face-to-face contact was reduced due to the early discharge the automated messages and the use of instructions videos were a suitable way for informing the new parents. This relates to Bandura's viewpoints on interactive computer-assisted feedback as a convenient means to inform, enable, motivate, and offer support [48]. It offers a way to reassure parents that their newborn is healthy and to help parents to feel in control of their new situation, which are factors that enhance a postnatal sense of security [12] as well as PSE [17, 18, 48] (Table 2).

Another aspect of the instruction videos is the potential of enhancing PSE through vicarious experiences, where the parents can see others perform, for instance, breastfeeding positions and bathing the child (Table 2).

The results revealed that the nurses feel the app enhances patients' curiosity and, to some extent, it encourages parents to act more independently, because they can easily search for information themselves. The nurses experienced that the new parents are more likely to seek for information themselves, when it is digitalized than in a paper pamphlet. According to Bandura, acting independently and thereby gaining one's own experience are a way of achieving mastery experiences, which strengthen PSE [18] (Table 2).

The nurses found that the automated messages serve to reassure parents, and this suggests that the messages could potentially have the effect of encouragement. According to Bandura, verbal persuasion contributes to PSE because the parents are convinced that they can cope successfully [18]. This can contribute to the achievement of a feeling of success. Also personal messages with encouraging feedback from healthcare professionals could to some extent substitute for the verbal persuasion that the families would receive if they were admitted for a longer duration after childbirth.

Bandura also states that because it is readily accessible and convenient, there are advantages in offering internet-delivered guidance. This is reflected in our study, where the nurses point out that the asynchronous communication is essential to their view of the app as a lifeline. It is easy to seek help; the families do not encounter a barrier in contacting the nurses for advice. This is because they do not feel that they are disturbing the nurses, as opposed to making a synchronous phone call. This is described in the literature as an issue in healthcare, because patients are often reluctant to contact healthcare professionals, even when they have something important to ask or discuss [2, 49]. It seems that the app has potential to be more efficient in ensuring access to healthcare than a phone.

Other studies have tested videoconferencing in the postnatal period [23, 24, 26]; it was valued as a supplement to traditional practice. The midwives saw that communicating via videoconferencing was almost equivalent to having a face-to-face meeting. The same was found in other studies that involved videoconferencing; the healthcare professionals experienced that it is possible to create an intimate relationship and proximity in technology-mediated care and that it provides a tool for patients to develop a sense of security at home [50, 51].

The transmission of photos gives new options compared to phone-mediated contact. A photo can “say more than a 1000 words” [52], where the nurses can actually see and observe instead of both families and nurses having to rely on written or oral descriptions over the phone. Other studies have pointed out further advantages for patients in staying at home instead of going to the hospital, in terms of time saved on travelling and waiting for a consultation [53].

The use of online communication such as e-mail or text messaging involves a language-analogue mediation—it is a dialogue, but not like a dialogue that two people have face-to-face or mediated by the phone [52]. The nurses addressed that the online chat function changed their way of communicating with the families, which they experienced to change their support to the new parents. This can be explained by applying Ihde's postphenomenological theory, where he underlines that the technological mediation of human practice shapes our experiences of the situations in which we are engaged. Technology is not a neutral tool; it provides a framework and invites us to employ certain use-patterns [52, 54–56]. When communicating face-to-face or on the phone, they felt they could use more of their senses to assess the patient's expressions or voice and evaluate their emotional or mental state as when communicating online. In this situation, as compared to when conducting a written dialogue, they felt it would be more natural for them to extend the dialogue to issues other than the one initially addressed.

However a report from the Institute for Healthcare Informatics [57] on the use of social media shows that patients also use social media for emotional support, which indicates that it is no longer only through face-to-face dialogue that people feel they can get emotional support. The report concludes that there have been essential changes in the way people communicate, and as a consequence the new technologies will change how healthcare operates on a global scale [57]. This development is also underpinned by a review by Plantin and Daneback [58] that showed the majority of today's parents search for not only information, but also social support on the internet. As a result of this development and because of the reduction in face-to-face contact, it has become more common for hospital staff to both communicate online [27, 59] and offer telephone support [60] following early discharge.

The limitation of our study is that it was a small-scale study. However the participatory design process with involving the participants in the design of the technologies was valuable. We could use the concrete experiences with the use of the app in the intervention in the further design process, where there had to be adjustments to the chat function. The new adjustments mean that the nurses do not have to check the computer for new messages, but they got an iPhone, where they receive a notification, whenever there is a new message.

There is a potential to assess the app in a randomized controlled trial for a more generalizable knowledge.

The development nurse on the ward was chosen to be the comoderator. She was newly employed and had not been a part of the intervention. Yet some of the nurses at the ward were familiar with her, which could contribute to a comfortable and safe atmosphere during the interview [35, 61].

## 7. Conclusion

The app gives the nurses the possibility to offer support and information to the parents being early discharged, as the app is experienced as a lifeline that connects the homes of the new parents with the hospital.

The written asynchronous communication provides an easy way for the nurses to offer the new parents support, when they are being early discharged, because the parents find it easier to contact the nurses through the app than the phone. This provides access to the healthcare professionals, which is essential in order to ensure parents' postnatal sense of security.

The automated messages are a suitable way for informing the new parents and it encourages them to act independently, which can enhance parental self-efficacy because the parents are inspired to take action thereby gaining mastery experiences.

The nurses experience that the app offers an efficient way to provide information to the parents as compared to pamphlets, because the parents were more likely to seek information when it was digitalized.

The nurses generally tend to focus their actions around providing information, and they do not consider that written communication lends itself to a more open and extended dialogue. This could be a question of needing more time to adapt to this new way of communicating. With more time, they could possibly use the asynchronous communication not only to convey information and for observation purposes, but also to offer emotional support.

## Figures and Tables

**Table 1:** Process of analysis, examples from the analysis.

Step 1: from medley to themes: superior themes extracted after the first open reading of the text	Step 2: from themes to codes. Identifying the meaningful units. The meaningful units are coded based on the superior themes as well as the preunderstanding and the theoretical frame		Step 3: from codes to meaning. The meaningful units are sorted into groups with respect to the codes; hereby overall categories arise from the coding process, which then are divided into subcategories
	Quotations	[Code]	
No tears	“I answer their questions… (…). I look at the photo of the umbilicus for instance or whatever it is. But I do not have the mother's tears. It creates a distance”	*[Lack of senses] *	Telemedicine as a means of providing support Telemedicine as a means for timely and accessible information.
		*[one sided dialoque] [sic] *	
Open door	“And I think that it is a help. They feel that it is ok that they take contact”	*[help available] *	
Repetition	“Then they get the pop-up messages which means they get the information one more time, that's great”	*[timely information] *	
A lot of information in a short time	“You are just talking, talking, talking… And how much do they really remember?”	*[Too much information] *	

**Table 2:** 

Functions of the app	Aspects supported	Supports
Knowledgebase, videos & information	Possibility for consistent relevant information	PPSS
	Acting independently (mastery experiences)	PSE/PPSS
	Seeing others perform, for instance, videos about breastfeeding (vicarious experiences)	PSE

Automated messages	Timely information	PPSS
	Being reassured (verbal persuasion)	PSE/PPSS

Online chat, asynchronous communication	Access to healthcare	PPSS
	Being reassured (verbal persuasion)	PSE
	Support (verbal persuasion)	PSE/PPSS
